# Temporal stability and maintenance mechanisms of alpine meadow communities under clipping and fertilization

**DOI:** 10.1002/ece3.8128

**Published:** 2021-10-28

**Authors:** Ting Wang, Chenglong Guo, Silin Sang, Yiting Liu, Gang Liu, Desheng Qi, Zhihong Zhu

**Affiliations:** ^1^ College of Life Sciences Shaanxi Normal University Xi’an China; ^2^ Key Laboratory of Medicinal Animal and Plant Resources of Qinghai‐Tibetan Plateau in Qinghai Province Xining China; ^3^ College of Life Sciences Qinghai Normal University Xining China

**Keywords:** clipping, fertilization, stability mechanisms, temporal stability

## Abstract

Negative effects of long‐term overgrazing have been seriously, grasslands temporal stability is an important ecological concern we need to research. Here, we performed a 12‐year‐long (2007–2018) two‐factor controlled experiment on *Kobresia humilis* meadow on the Tibetan Plateau. The manipulations included three clipping levels (no clipping, NC; moderate clipping, MC; heavy clipping, HC) and two fertilization levels (no fertilization, NF; fertilization, F). Our results revealed that the two clipping manipulations significantly increased the temporal stability of alpine meadow communities, whose significant increase was more pronounced under the MC than HC treatment. Species asynchrony had a significant positive correlation with species abundance along with compound community gradient. Moreover, asynchrony effects, portfolio effects, and facilitation interactions were all present in the communities under the six types of experimental treatment combinations. Additionally, a selection effect was detected in the compound communities, demonstrating characteristics that are common to different mechanisms. There were no significant differences in the effects of these mechanisms on community temporal stability between the NC–NF and MC–NF interactive communities. The portfolio effects predominated when clipping intensity was moderate under both fertilization and nonfertilization conditions. By contrast, in the compound communities, the selection effect predominated. In summary, we conclude that in meadow communities that undergo clipping and fertilization disturbances, facilitation interactions and weak interactions make a greater contribution toward maintaining their temporal stability.

## Graphical abstract

Under six experimental conditions of clipping and fertilization levels, we found that fertilization, as well as heavy and especially moderate clipping intensity, significantly increased the temporal stability of *K. humilis*. Asynchrony effects, portfolio effects, and facilitation interactions were all present under the six experimental treatment conditions in this study. Clipping mainly increased community temporal stability via the asynchrony effect, whereas this increase under fertilization treatment was mainly owing to facilitation and weak interactions.

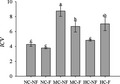

## 1 INTRODUCTION

Generally, temporal stability of a community of organisms is expressed by using the inverse coefficient of variation (ICV) of ecosystem functional markers at the time scale (Cardinale et al., 2013; Gross et al., 2014). The higher the ICV, the greater the temporal stability. Therefore, increasing a given ecosystem function and decreasing any effects on its temporal variability should increase temporal stability. Many studies have shown that increasing species diversity or species abundance can increase ecosystem function and decrease its variability, thereby increasing temporal stability (Cardinale et al., 2013). Clipping and fertilization were the most important management practices in global grassland ecosystems (Connolly et al., 2013). Studying stability benefited to reveal grasslands response intensity and its mechanism to clipping and fertilization disturbance (Tilman, 1999).

Currently, six leading mechanisms or theoretical hypotheses are invoked to explain temporal stability mechanisms for global grasslands species abundance: *Mechanism* *1*: asynchrony effect, which refers to an increased species abundance leading to interspecific competition or negatively correlated responses of species toward environmental changes that decrease interspecific covariance or promoted greater and greater negative covariance (Thibaut & Connolly, 2013). This results in stable effects on the community due to interspecific complementary dynamics (Bai et al., 2004; Downing et al., 2014; Grman et al., 2010); *Mechanism 2*: portfolio effect, such that when the species evenness of a community is high and its total biomass is constant, if increasing the species abundance causes random species fluctuations, this causes species biomass variance *σ* ^2^ to decrease faster than the mean species biomass *m*, which follows the power function *σ* ^2^ *= cm^z^* and *z* > 1, so that the community's stability is strengthened overall (Hillebrand et al., 2008; Mariotte et al., 2013; Roscher et al., 2011); *Mechanism 3*: over‐yielding effect, which occurs when increased species abundance causes greater interspecific functional complementation, which increases the use efficiency of species for limited resources, thus augmenting the biomass of low‐productivity species to increase ecosystem function *μ*, eventually driving *m* to increase faster than its *σ* and thus producing stable effects (Tilman, 1999); *Mechanism 4*: selection effect, also known as the dominance effect, wherein stable effects are produced by stable and dominant high‐productivity species in the community (Hillebrand et al., 2008). Whether the selection effect can increase temporal stability depends on the stability of one or more dominant species (Zhou et al., 2005), so it is ultimately not related to or negatively correlated with community‐level species abundance (Roscher et al., 2011; Yang et al., 2011). Many studies have emphasized the importance of disturbance or environmental changes in causing communities to switch from a high diversity (low dominance) stable state to a low diversity (high dominance) stable state (Deutschman, 2001; Tilman, 1999; Wilsey et al., 2014); *Mechanism 5*: facilitation interactions, which mainly arise as direct, non‐nutritional positive interactions produced by certain species upon other species by regulating and improving the physical environment of the community, or the stable effect produced when crucial resources are provided to other co‐occurring species, and it is not associated with niche differentiation (Deutschman, 2001; Isbell et al., 2009); and *Mechanism 6*: weak interactions, whereby only consumer stress is increased in the community. This shifts strong, nutrient competitive relationships between low trophic level species to a weak trophic interrelationship and weakens interspecific disturbance, thereby increasing population and community temporal stability (Deutschman, 2001; Proulx et al., 2010). To sum up, grasslands stability mechanism seems from asynchrony effect under clipping and fertilization at both species and functional group levels (Chu et al., 2020; Downing et al., 2014).

The above six mechanisms can be subsumed under “complementary” effects (Mechanisms 1, 2, 3, 5, and 6, based on species niche differentiation, interspecies complementation or facilitation) and “selection” effect (Mechanism 4, based on the dominance of a few species in the community). No study has found that evidence of these mechanisms can simultaneously operate in a specific community, and only a few of these mechanisms have been found to occur simultaneously, such as the overyielding, asynchrony, and portfolio effects (Lehman & Tilman, 2000); asynchrony and portfolio effects, and facilitation or weak interactions (Yang et al., 2012); or portfolio, asynchrony, and dominance effects (Isbell, Reich, et al., 2013; Isbell et al., 2013). Since species diversity and community dominance usually have opposing trends in variation, it follows that the complementary effects caused by increased species diversity and the selection effect caused by stable dominant species are mutually exclusive (Wilsey et al., 2014; Yang et al., 2011). In addition, the portfolio effect should be present in a community, regardless of whether the other mechanisms are present or absent (Wilsey et al., 2014).

Existing studies were conducted in North American Prairie and Eurasia Steppe, and grassland ecosystem stable mechanisms were not clear under clipping and fertilization management in alpine meadow. This study was carried out on Tibetan Plateau. Three major ecological questions were addressed: (a) What are the relative effects of clipping and fertilization on the productivity and temporal stability of alpine meadow communities? (b) What mechanism(s) that can maintain temporal stability are present in communities under the different clipping and fertilization treatments? And how do these mechanisms change with clipping intensity and fertilization levels?

## 2 MATERIALS AND METHODS

### 2.1 Overview of the study site

This study was conducted at the Chinese Academy of Sciences Haibei National Field Research Station of Alpine Grassland Ecosystems. This station is located in Menyuan Hui Autonomous County, the Haibei Tibetan Autonomous Prefecture, Qinghai Province; its geographical coordinates are 37°29′–37°45′N and 101°12′–101°23′E, and its elevation is 3,220 m (Zhao et al., 2008). The station has an alpine climate characterized by not only low temperatures and thus low accumulated temperature but also short warm seasons and long cold seasons. Its mean annual temperature is only −1.7°C, and the mean temperature of the coldest month is −15°C and that of the hottest is 10.1°C, with low annual temperature variation but high diurnal temperature differences. There is no absolute frost‐free period during the entire year, and the relative frost‐free period is just 20 days long (Li et al., 2004). The mean annual precipitation at the station is 560 mm, most of which (79%) comes in June–September. In addition, sunlight is abundant at the study site and solar radiation is strong: Mean annual solar radiation is 25:00–36:00 hr and total annual radiation is 5.0 × 10^6^–6.0 × 10^6^ kJ/m^2^ (Li et al., 2004; Zhao et al., 2008). The site mainly consists of alpine meadow soil, young developing soil, and sparse vegetation (Zhao et al., 2008). Due to the effects of low temperature, the organic matter and humus content of the surface soil layer are higher, at ca. 6%–12%; however, the soil layer is thin and soil pH is neutral or weakly acidic (6.0–7.5) (Zhou & Wu, 2006). The sampling plot (6,000 m^2^) was situated in the classical *Kobresia humilis‐*dominated meadow ecosystem. The terrain of this meadow is flat, having a soft grass quality, abundant nutrients, and high calorific value, making it an crucial grazing pasture for animals during winter and spring (Pu et al., 2005). Other types of alpine meadows occur at the study site: Besides the *Kobresia humilis* meadows on flat beaches, there are *Kobresia pygmaea* meadows on sunny slopes, the *Kobresia tibetica* swamp meadow on both banks of rivers, and *Potentilla fruticosa* shrub meadows established on shady and semi‐shady slopes.

### 2.2 Study methods

#### 2.2.1 Experimental design

The experiment's sampling area, constructed in late April 2007, was 100 m long and 60 m wide, and enclosed by barbed wire. This sampling area was situated on flat terrain previously grazed as pasture for domestic yak and Tibetan sheep in winter and spring before its construction. Continuous grazing occurred during winter and spring, with breaks in summer and autumn. The annual pasture utilization rate was ca. 45%–50%, considered to be a moderate grazing intensity. A two‐factor, split‐plot design was used, for which the whole plots received the clipping treatment and their subplots received the fertilization treatment. Thus, subplots were nested in the whole plots. The sampling area was divided into five types of blocks (of which blocks I, III, and V were used for community surveys and blocks II and IV to measure net primary productivity in the experimental communities). In each block, five 4 m × 4 m whole plots were set up, and each block was replicated thrice (so 15 blocks in total).

Three clipping levels were applied to the whole plots (Figure S1), consisting of plant stubble heights of 1, 3 cm, and no clipping, which corresponded to approximately 60%–70%, 45%–50%, and 0% of the total biomass removed, respectively. In other grazing intensity experiments conducted on this type of pasture (Zhu & Wang, 1996; Zhu et al., 1994, 2012), pasture utilization rates for heavy and moderate grazing were 60% and 45%, respectively; hence, the three clipping levels applied may be reliably used to simulate heavy grazing (HC), moderate grazing (MC), and no grazing (NC). For the subplot fertilization treatment, four 2‐m‐long and 0.25‐m‐wide galvanized zinc boards were buried at a depth of 0.25 m in a “cross” shape, so that each whole plot was divided into four 2 m × 2 m subplots, to which two fertilization (F) and two nonfertilization (NF) treatments were applied (Figure S1). These metal boards prevented the lateral entry of water from adjacent fertilized subplots and avoided fertilization from affecting other subplots. Fertilization was carried out in mid‐May, mid‐June, and mid‐July of every year. During each treatment, 4.60 g/m^2^ urea (containing 20.4% nitrogen) and 1.10 g/m^2^ diammonium phosphate (5.9% nitrogen, 28.0% phosphorus) were added. In 2007–2018, 13.80 g/m^2^ urea and 3.30 g/m^2^ diammonium phosphate were used yearly on average, and the total annual net amount of nitrogen and phosphorus added was 3.01 and 0.92 g/m^2^, respectively; these are higher than the optimal fertilizer used for the local construction of artificial grasslands (2.25 g/m^2^ N (Qiao et al., 2006)). A 1.5 m × 1.5 m area in each subplot's center was used for data measurements, and within each such area, four quadrats were set up: One was the permanent quadrats, for the measurement of vegetation species coverage, number of individuals, plant height, and total community coverage; the remaining three were sampling quadrats, used to measure plant traits and net primary productivity of the community (Figure S1).

#### 2.2.2 Plant sampling

Community surveys were conducted in the permanent subplots in blocks I, III, and V in mid‐August of every year in 2007–2016. Total community coverage, number of species and their respective coverage, plant height (20 individuals measured per plot, and all plants were measured if the number was <20), and number of individuals (number of individuals for dicotyledon plants, and number of plants for monocotyledons) per species were measured. A total of 108 plots were used for this sampling.

#### 2.2.3 Data calculations

##### Temporal stability (ICV)

$$\text{ICV}=\frac{\mu }{\sigma }=\frac{\sum \text{Cover}}{\sqrt{\sum \text{Var}+\sum \text{Cov}}}$$

Here, coverage was used to calculate the ICV. In the above equation, *μ* or ∑Cover is the temporal mean of total community coverage and *σ* is its temporal standard deviation. ∑Var represents the summed temporal variances for various species’ coverage in the community and ∑Cov represents the summed temporal covariances for various species’ coverage in the community. A greater ICV indicated higher temporal stability of the community.

##### Asynchrony effect

The asynchrony effect of the co‐occurring plant species was estimated using 1−*φ_b_*, for which *φ_b_* refers to the synchrony of species' fluctuations.

$$1‐\varphi_{b}=1‐\frac{\sigma^{2}}{{\left(\sum_{i=1}^{S}\sigma_{i}\right)}^{2}}.$$

In the equation above, *σ* ^2^ represents the temporal variance of total community coverage and *σ_i_* is the temporal standard deviation for coverage the *i*th species in the community, for which 0 ≤ 1−*φ_b_* ≤ 1. If 1−*φ_b_* = 1, complete asynchrony is present, if 1−*φ_b_* = 0, complete synchrony is present.

##### Portfolio effect

Twenty years ago, Tilman (1999) proposed a power function describing the temporal variance of intracommunity species abundance and their mean abundance to determine whether the portfolio effect is present.

$$\sigma^{2}=cm^{z}$$

The logarithm of both sides of the equation is obtained to derive:

$$\log\left(\sigma^{2}\right)=z\times \log\left(m\right)+\log\left(c\right)$$

In the above equation, *σ* ^2^ represents the temporal variance of species coverage and *m* represents the temporal mean of species coverage. If *z* > 1, then the portfolio effect is present in that community and the total variance of species community decreases as species diversity increases.

##### Species importance value (IV)

The dominant species in the various experimentally treated communities were determined according to the IV values, in this way:

IV=(relative density+relative plant height+relative biomass)/3

##### Species abundance (SR)

This was simply represented by the number of species found in the sampled quadrat plots.

#### 2.2.4 Statistical analysis

Before analysis, testing for normal distribution and variance homogeneity in data was first carried out. Data that did not fulfill either assumption were transformed before the statistical analysis was carried out. The significance level for inferential statistics was set to *p* < .05.

If interaction was present between clipping and fertilization on the response variables, one‐way ANOVAs were used to compare the 6 different treatment combinations. Univariate ANOVA (two‐way, crossed) was used to test for significant differences in the effect values (*φ_b_*, ∑Var, CV_Dom_, and CV_Pop_) for the same temporal stability under the different clipping and fertilization treatments. Duncan's multiple range test was used for multiple comparison of mean ICV values and temporal stability mechanism effects between the different treatments.

The SPSS (20.0) was used for linear regressions of the data, with the *F* test used to test for significance of the slope (*p* < .05) and the highest *R* ^2^ value to determine the best fitting regression model. To validate the relative contribution of different temporal stability mechanisms on community temporal stability, stepwise regression analysis was performed. The standardized partial regression coefficient was used to test for relationships between a possible asynchrony effect (synchrony *φ_b_*), portfolio effect (∑Var), selection effect (CV_Dom_ of dominant species), facilitation interactions, and weak interactions—mean coefficient of variation for the population, CV_Pop_—in each of the six combined‐treatment plant communities and overall community CV_Com_, and to determine the relative importance of different temporal stability mechanisms operating in the meadow community by R statistic.

## 3 RESULTS

### 3.1 Effects clipping and fertilization on the temporal stability of alpine meadow communities

**FIGURE 1:**
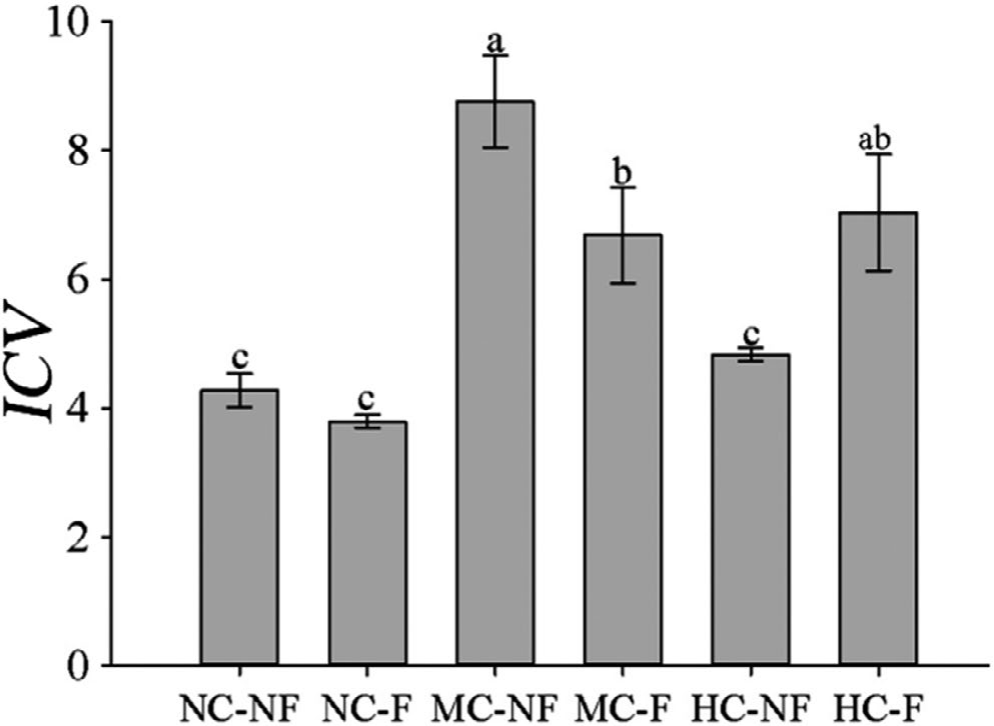
Effects of clipping and fertilization on ICV of communities (mean ± *SE*). Notes: NC‐NF, NC‐F, MC‐NF, MC‐F, HC‐NF, and HC‐F indicate no clipping and fertilization, fertilization, moderate clipping, moderate clipping and fertilization, heavy clipping, heavy clipping, and fertilization, respectively. ICV is temporal stability of community. Same letter above the standard error bars indicates no difference among treatments (*p* > .05), and different letters indicate significant differences among the treatments (*p* < .05)

ANOVA results showed that clipping and its interaction with fertilization significantly affected community temporal stability (*p* < .05), whereas fertilization alone did not significantly affect it (Table 1). This result conforms to the moderate disturbance hypothesis: In that, clipping increased community temporal stability differently between F (fertilized) and NF (nonfertilized) communities (Figure 1). Both moderate (MC) and heavy clipping (HC) increased community temporal stability by 95.26% and 49.38% compared with control. Meanwhile, MC is approximately 30.72% higher than HC.

**TABLE 1:** Univariate ANOVA for the effects of clipping and fertilization treatments on the temporal stability of meadow plant communities

Source of variance		*df*	ICV	
			*F*‐test	*p*
Whole plot	C	2,4	25.893	.005
	B	2,4	2.413	.205
	B × C	4,6	0.951	.496
Subplot	F	1,6	0.073	.796
	C × F	2,6	8.492	.018

Note

B, block; C, clipping; *df*, degrees of freedom; F, fertilization; ICV, temporal stability; ×, interaction.

### 3.2 Tests on different clipping and fertilization community temporal stability maintenance mechanisms

#### 3.2.1 Asynchrony effect

**FIGURE 2:**
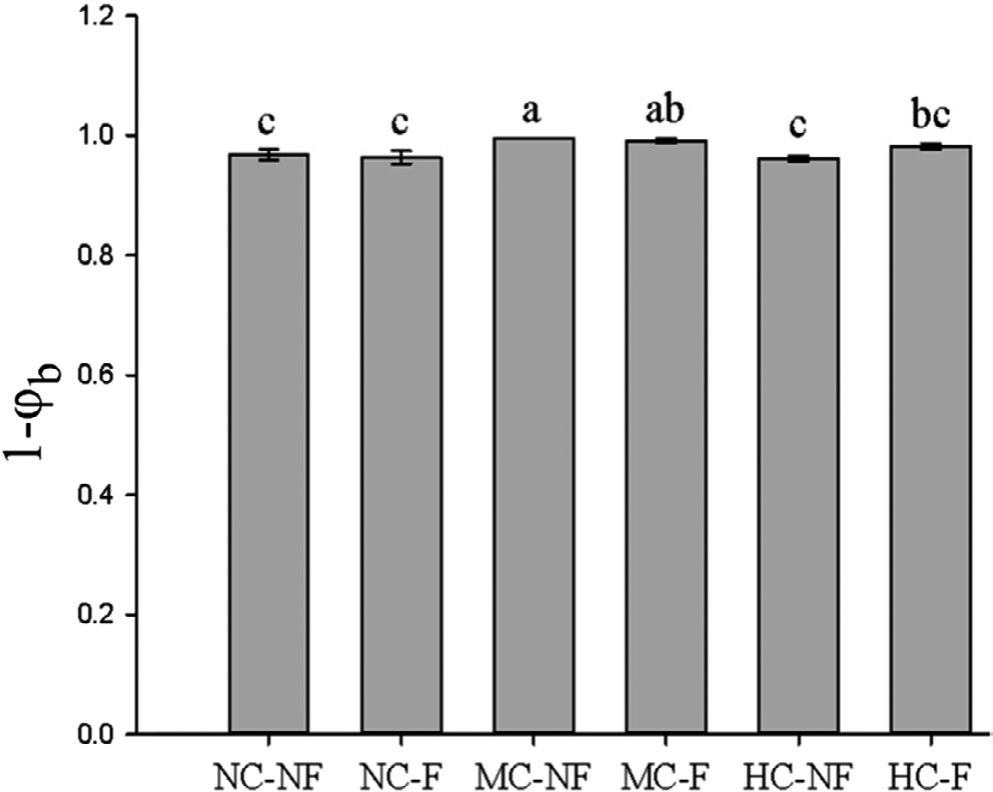
Effects of fertilization and the interaction between clipping and fertilization on the species asynchronism of communities (mean ± *SE*). Notes: NC‐NF, NC‐F, MC‐NF, MC‐F, HC‐NF, and HC‐F indicate no clipping and fertilization, fertilization, moderate clipping, moderate clipping and fertilization, heavy clipping, heavy clipping, and fertilization, respectively. 1−*φ*_b_ expressed the degree of species asynchronism. Same letter above the bars indicates no differences among the treatments (*p* > .05), and different letters indicate significant differences between them (*p* < .05)

The obtained 1−*φ_b_* values for the six different treatments were all <1, showing that the species fluctuations in these communities were not synchronous and the asynchrony effect was present in each. Regressions revealed species asynchrony along with the compound community gradient (1−*φ_b_*) had a significant positive correlation with species abundance (SR) (*R* ^2^ = .205, *F*_(1,106)_ = 27.339, *p* < .001, (1−*φ_b_*) = 0.001 SR + 0.892); hence, the asynchrony effect decreased as species abundance increased. According to Table 2, the *p*‐values for B × C and F terms are .086 and .538, respectively, showing that the effects of the interaction between group, and clipping treatment and fertilization on 1−*φ_b_* are not significant (*p* > .05). The corresponding *p*‐values for C, B, and C × F were .05, .049, and .07, respectively (*p* <.05), showing that clipping, group, and clipping ×fertilization had significant effects on the 1−*φ_b_* values. As Figure 2 shows, asynchrony increases as clipping intensity increases and was greatest under moderate clipping of vegetation. Clipping increased the asynchrony differences between F and NF meadow communities (Figure 2).

**TABLE 2:** Univariate ANOVA for the effects of clipping and fertilization treatments on the asynchronization of species coverage in meadow plant communities

Source of variance		*df*	Asynchronism	
			*F*‐test	*p*
Whole plot	C	2,4	25.908	.005
	B	2,4	7.026	.049
	B × C	4,6	3.453	.086
Subplot	F	1,6	0.426	.538
	C × F	2,6	12.674	.007

Note

B, block; C, clipping; *df*, degrees of freedom; F, fertilization; ×, interaction.

#### 3.2.2 Portfolio effect

The power function *σ* ^2^ = *cm* ^z^ was used for the log(*σ* ^2^) versus log(*m*) regression, that is, log(*σ* ^2^) = *z* × log(*m*) + log(*c*). The estimated regression coefficient *z* shows the rate by which log(*σ* ^2^) changes per unit change in log(*m*) and the regression constant is log(*c*). These regressions revealed that the total variance of species coverage along with the community gradient for the six treatment combinations and their mean coverage was positively correlated in a significant way (Table S1, *p* < .001). The *z* values for the six communities all exceeded 1 (i.e., between 1.545 and 1.733; Table S1), showing that the portfolio effect was present in the different experimental community combinations of clipping and fertilization treatments. Regressing species ∑Var at the compound community gradient against species abundance (SR) showed positive relationship between the two variables (*R* ^2^ = .078, *F*_(1,106)_ = 9.006, *p* = .003, ∑Var = 17.958SR−342.555).

**FIGURE 3:**
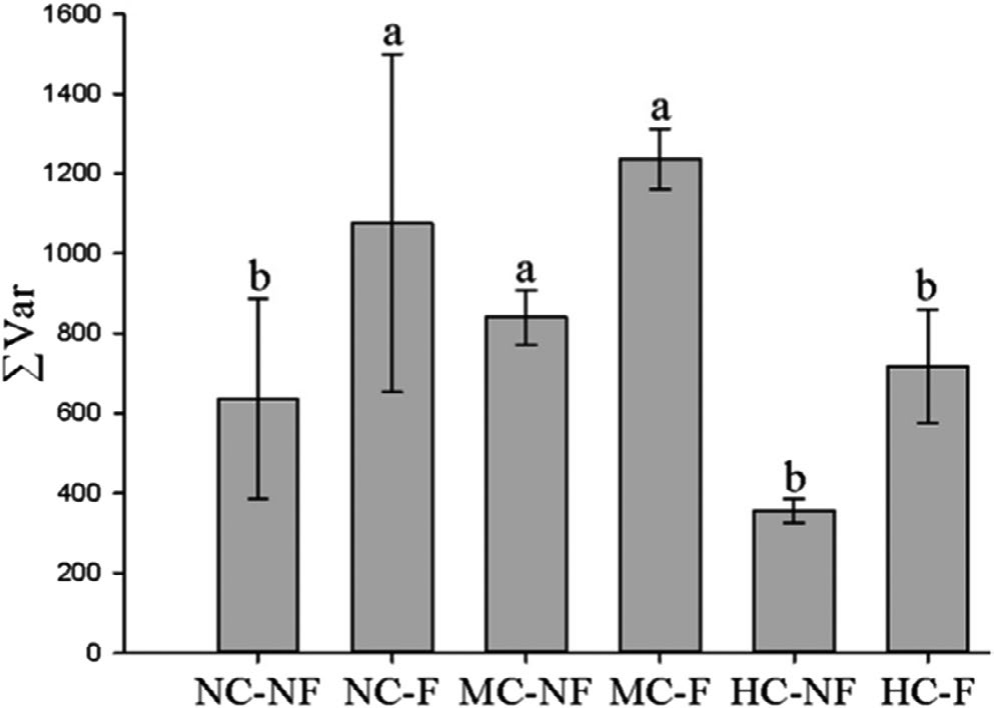
Effects of fertilization and the interaction between clipping and fertilization on the species portfolio effect of communities (mean ± *SE*). Notes: NC‐NF, NC‐F, MC‐NF, MC‐F, HC‐NF, and HC‐F indicate no clipping and fertilization, fertilization, moderate clipping, moderate clipping and fertilization, heavy clipping, heavy clipping, and fertilization, respectively. ΣVar represents the portfolio effect. Same letter above the bars indicates no differences among the treatments (*p* > .05), and different letters indicate significant differences between them (*p* < .05)

The ANOVAs showed that clipping, fertilization, and clipping × fertilization have significant effects on community portfolio effects (Table 3). Clipping and fertilization increased ∑Var, while clipping decreased the ∑Var differences between the F and NF communities (Figure 3).

**TABLE 3:** Univariate ANOVA for the effects of clipping and fertilization treatments on the portfolio effects of community coverage

Source of variance		*df*	Portfolio effect	
			*F*‐test	*p*
Whole plot	C	2,4	13.013	.018
	B	2,4	1.184	.394
	B × C	4,6	0.848	.543
Subplot	F	1,6	9.017	.024
	C × F	2,6	6.052	.036

Note

B, block; C, clipping; *df*, degrees of freedom; F, fertilization; ×, interaction.

#### 3.2.3 Selection effect

The species importance value (IV) was used to determine the dominant species present in the communities formed by the six different treatment combinations regardless of changes in clipping intensity, the dominant species for NF community was always *Stipa aliena,* and the dominant species for the F community was *Elymus nutans*.

Table 4 showed the dominant species (R) for the six treatment communities (i.e., NC‐NF, NC‐F, MC‐NF, MC‐F, HC‐NF, and HC‐F). First, we fit a regression for the coefficient of variation of the dominant species in various communities (CV_Dom_) and community species abundance (SR) at the temporal scale to determine whether dominant species stability was independent of species abundance. It was showed that in both the NC‐NF and MC‐F communities, SR and CV_Dom_ were significantly related (Table S2), while dominant species stability was independent of species abundance in the other four communities (*p* > .05) and the explanatory power of CV_Dom_ for the SR changes was 0%–28.6%. These results indicated the selection effect might be present in the NC‐F, MC‐NF, HC‐NF, and HC‐F communities.

**TABLE 4:** Dominant plant species of different experimental meadow communities

Ranking	NC‐NF	NC‐F	MC‐NF	MC‐F	HC‐NF	HC‐F
R	*Stipa aliena* (0.20)	*Elymus nutans* (0.20)	*Stipa aliena* (0.12)	*Elymus nutans* (0.30)	*Stipa aliena* (0.11)	*Elymus nutans* (0.39)

Note

Abbreviations NC, MC, HC, respectively, indicate no clipping, moderate clipping, and heavy clipping, while NF and F indicate the no fertilization and fertilization treatments. R denotes the dominant species. R represents the first‐ranked species in each of the communities.

Regression (Table S3) was also applied to the CV_Dom_ of dominant species of the various communities and the coefficient of variation for community species coverage (CV_Com_). No linear relationships were found between CV_Dom_ and CV_Com_ in the six communities (*p* > .05), but a negative one was detected for the CG community (*p* < .05), which showed that the selection effect is present in the community.

#### 3.2.4 Facilitation interactions and weak interactions

Regressions were performed between the mean coefficient of variation for various species at the compound community gradient (CV_Pop_) and species abundance (SR) at the temporal scale. This showed CV_Pop_ and SR as positively correlated (*R* ^2^ = .03, *F* = 3.266, *p* = .074, *y* = 0.003*x* + 1.658). The explanatory power of SR for changes in CV_Pop_ was just 3%. Therefore, according to this evidence, facilitation interactions and weak interactions were not present in the six experimental communities.

**FIGURE 4:**
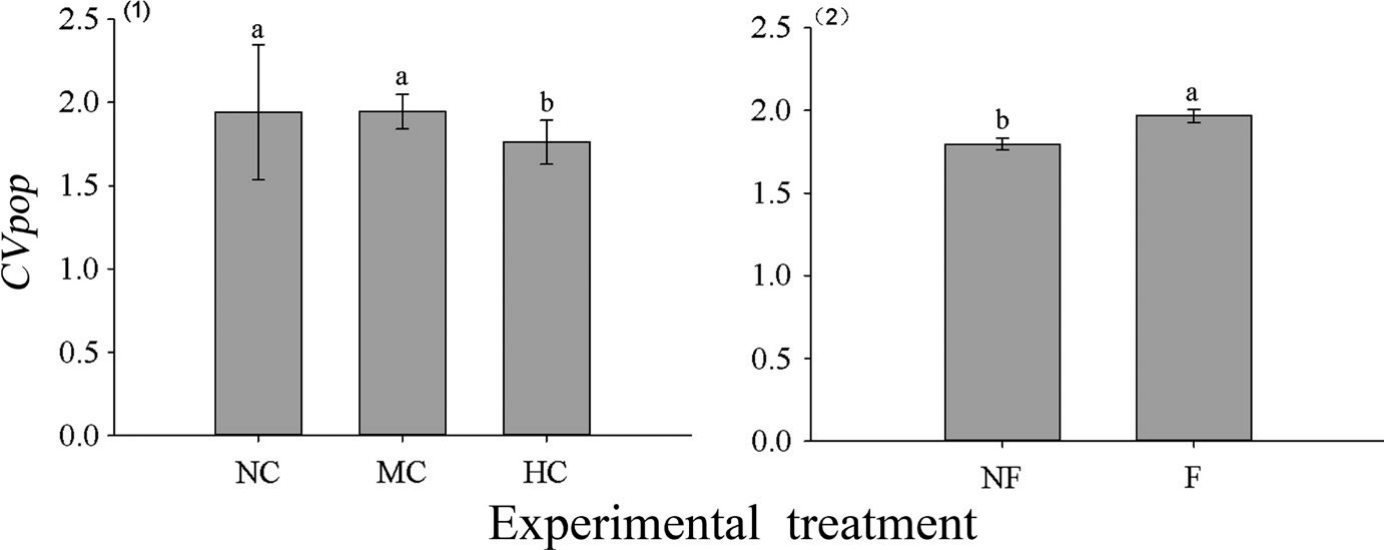
Effects of fertilization on the CV_Pop_ (mean ± *SE*) of meadow communities. Notes: NF and F indicate no fertilization and fertilization, respectively. CV_Pop_ is mean coefficient of variance of the population. Same letter above the bars indicates no differences among the treatments (*p* > .05), and different letters indicate significant differences between them (*p* < .05)

The ANOVAs showed that clipping and fertilization independently affected CV_Pop_, with a nonsignificant interaction found (Table 5). Clipping reduced the mean CV_Pop_, while fertilization increased it (Figure 4).

**TABLE 5:** The results of the univariate ANOVA for clipping and fertilization treatments on CV_Pop_

Source of variance		*df*	CV_Pop_	
			*F*‐test	*p*
Whole plot	C	2,4	8.816	.034
	B	2,4	1.649	.300
	B × C	4,6	4.752	.045
Subplot	F	1,6	83.868	<.001
	C × F	2,6	0.547	.605

Note

B, block; C, clipping; *df*, degrees of freedom; F, fertilization; ×, interaction.

### 3.3 Relative contributions of different mechanisms on community temporal stability

**FIGURE 5:**
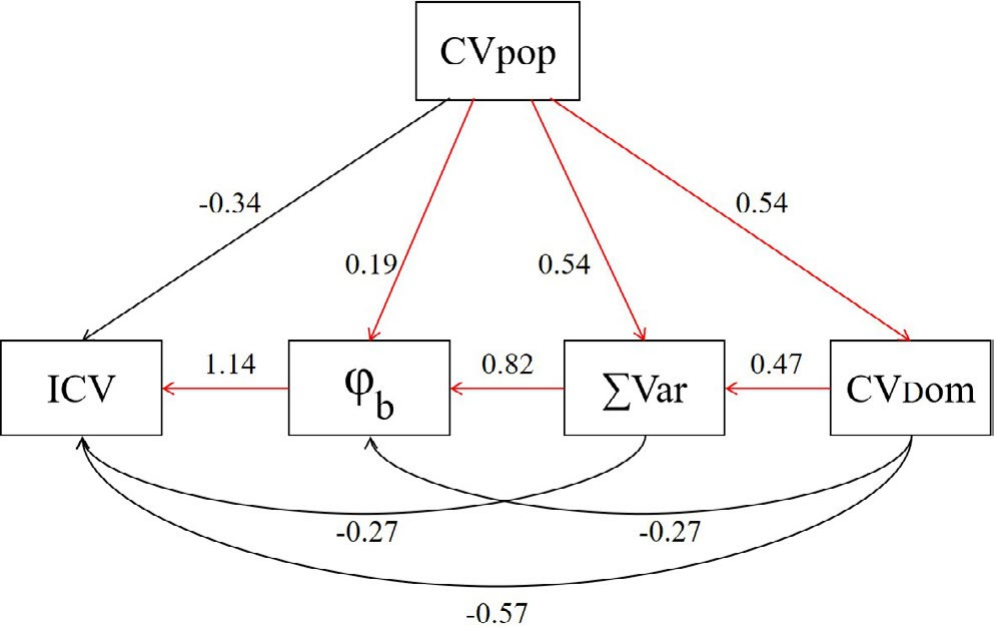
Path analysis of the temporal stability and maintenance mechanisms in meadow communities. Note: ICV is temporal stability of community. *φ_b_* represents the degree of species synchronism. ΣVar represents the portfolio effect. CV_Dom_, coefficient of variance of dominant species. CV_Pop_, mean coefficient of variance of the population. Fisher's C = 10.425 with *p*‐value = .108

Path analysis showed the relationships between clipping and fertilization treatments, species diversity changes, temporal stability mechanisms, and population and community temporal stability (Figure 5). Based on the within factor range examined, the community temporal stability (ICV) was directly affected by four factors. The asynchrony effect (*φ_b_*) was positively correlated with community temporal stability. Conversely, the portfolio effect (∑Var), selection effect (CV_Dom_), and facilitation interactions and weak interactions (CV_Pop_) were all negatively correlated with community temporal stability. The selection effect (CV_Dom_) was negatively related to the asynchrony effect (*φ_b_*), while the portfolio effect (∑Var) was positively correlated with facilitation interactions and weak interactions (CV_Pop_), as well as with the asynchrony effect (*φ_b_*). Asynchrony effect (*φ_b_*), selection effect (CV_Dom_), and facilitation interactions and weak interactions (CV_Pop_) were all positively correlated with portfolio effect (∑Var); likewise, the portfolio effect (∑Var) and facilitation interactions and weak interactions (CV_Pop_) were both positively correlated with selection effect (CV_Dom_). Asynchrony effect (*φ_b_*), portfolio effect (∑Var), and selection effect (∑_Dom_) were positively correlated with facilitation interactions and weak interactions (CV CV_Pop_). Among these relationships, the relationship between community temporal stability and asynchrony effect was the strongest.

## 4 DISCUSSION

### 4.1 Effects of clipping and fertilization on the temporal stability of alpine meadow communities

Many studies demonstrate that more community species diversity promotes facilitation interactions that increase the functional stability of the ecosystem (Cardinale et al., 2013; Isbell et al., 2011; Tilman, 1999). Our study showed that moderate and heavy clipping increased the temporal stability of alpine meadow communities. This effect has also been verified in typical Inner Mongolian grasslands (Hillebrand et al., 2008) in China and by our previous studies (Wang et al., 2013; Yao et al., 2016). This is mainly because clipping induces competitive release effects in the community of species, ensuring the survival of a larger number of neighboring species as well as normally rare species (Isbell et al., 2009). This reduction of a competitive advantage in tall plants after clipping increases local species evenness (Bakker & Olff, 2003), which ultimately increases community species diversity. For example, Hooper et al. (2005) found that long periods of grazing (or clipping) disturbance were able to greatly affect species diversity, by releasing a large area of survival space and increasing resource spatial heterogeneity, thereby reducing competition between species for light resources and shifting competition among plants for light to competition for underground nutrient and water resources, which ultimately increased species diversity in the community. Furthermore, these spaces released by clipping may also provide suitable conditions for species occupying the same niche to enter, whose successful entry can also increase species diversity (Pan et al., 2015).

In addition, both the inhibitory effects and competitive release effects of grazing or clipping on competing dominant species can also lead to diversification in key functional characteristics in the community's members, which facilitates the maximum utilization of a set of limited resources by co‐existing species via different means (Hooper, 1998; Li et al., 2011). Therefore, increased species diversity and increased interspecific functional complementation promote overall community stability (Yao et al., 2016). Furthermore, we can see from the calculation for temporal stability that relative to species variance and covariance, intensive disturbance that perhaps increases the mean total community coverage will increase the temporal stability of communities (Yang et al., 2011). Our previous studies showed that the over‐compensatory plant growth after clipping (Xi et al., 2010) increased community‐level productivity (Pan et al., 2015). Consistent with this, in our current study, we found experimental evidence that clipping also simultaneously increases the temporal stability of communities in addition to increasing the species diversity of alpine meadow communities.

Although clipping had significant effects on community temporal stability, the fertilization did not significantly affect stability in this study. Many studies have shown that the asynchrony of species fluctuations is positively correlated with community temporal stability (Grman et al., 2010; Mariotte et al., 2013; Roscher et al., 2011). This is because as species abundance increases, interspecific competition will decrease interspecific covariance or result in higher negative variance that fosters interspecific complementation; that is, a reduction in the abundance of one species can increase the abundance of another species via compensation. The stronger the interspecific complementation, the higher the relative stability of the community (Mariotte et al., 2013; Roscher et al., 2011; Yang et al., 2012). The results of our study show that clipping increased species asynchrony (Table 2), which is related to the increase in community species abundance caused by clipping plants. Some studies reported that fertilization reduced the asynchrony of communities’ species, which decreased their temporal stability (Chen et al., 2016). However, through experimentation, we found that the effects of fertilization on the asynchrony effect were not significant (Table 2), thus showing that the asynchrony of species coverage itself cannot adequately explain the stability of community coverage. In our path analysis, the asynchrony effect was selected by model, being positively associated with community temporal stability. Hence, when studying community stability—a problem that includes complex interactions and relationships—path analysis offers a way convey and disentangle the complex linear relationships between multiple independent variables and dependent variables. The path model obtained can truly reflect the outcomes of these complex interactions and relationships. The reason why no significant linear relationship was found between asynchrony and community temporal stability in our one‐way regression analysis may be due to the presence of some limitations when we used coverage as a study marker. This is likely because the visual estimation error for plant species coverage may be larger. Yang et al. (2011) encountered similar problems when using coverage as asynchrony effect marker in their work.

Concerning the portfolio effect, the magnitude of the z value in the regression points to the potential positive effects of this mechanism upon species diversity and community temporal stability. Theoretically, as diversity increases, community temporal stability will decrease when *z* is <1 but increase when *z* is >1. In our study, the *z* values of all six types of clipping and fertilization combinations were greater than 1 (Table S1) and their population temporal stability and community temporal stability show significant positive correlations, which supports Tilman's theoretical study on *z* values.

### 4.2 Mechanisms of temporal stability in different experimental treatment communities

In this study, six different field experimental communities were produced from 12 years of treatments arising from the combination of three clipping intensities crossed with two nested fertilization levels (i.e., 3 × 2 factorial arrangement): NC‐NF, NC‐F, MC‐NF, MC‐F, HC‐NF, and HC‐F. Due to the long‐term effects of differing clipping intensity and fertilization levels, and their combinations, the temporal stability of these six community coverage values was different (Table 1). Long‐term clipping and fertilization disturbance also simultaneously caused changes in the mechanisms maintaining community temporal stability: In other studied communities, because clipping intensity increases species diversity (Kong et al., 2016) and species evenness (Bakker & Olff, 2003), the co‐existing species tend to fluctuate independently (Downing et al., 2014), thus allowing for significant complementary effects—including asynchrony and portfolio effects, and facilitation interactions—to operate in such communities. In communities, fertilization as a localized disturbance reduces community species diversity (Kong et al., 2016) and increases community dominance (Wang et al., 2013; Yang et al., 2011; Zhou et al., 2011), so that stable highly productive dominant species provide long‐term stabilizing effects (Yang et al., 2011), which would generate significant selection effects in the community.

In our regressions, as species abundance increased, total community species variance also increased. Therefore, the presence of the portfolio effect in communities was able increase community stability. The mean population coefficient of variation and species abundance also showed positive relationships, suggesting that the former's increase with the latter tended to make the community more stable. Nonetheless, we also obtained evidence that at species higher the species abundance, the greater was the asynchrony effect in the meadow communities, which agrees with many previous studies (Bai et al., 2004; Grman et al., 2010).

### 4.3 Different temporal stability mechanisms and driving effects of disturbance factors

Among the six different experimental communities, the asynchrony effect emerged as the main mechanism leading to increased community temporal stability under the clipping treatment. Clipping markedly increases species abundance and species diversity in plant communities (Grman et al., 2010) for which total species variance is then positively correlated with species abundance; that is, the sum of species variance increases with species abundance, and therefore, community stability is promoted.

In the NC‐NF, MC‐NF, and HC‐NF communities, although the asynchrony effect and portfolio effect were present, the respective effects of these mechanisms on community stability were similar. Therefore, we suggest these mechanisms are not major mechanisms that maintain community temporal stability in alpine meadow communities. However, under different fertilization treatments, the asynchrony effect and portfolio effect are major mechanisms that lead to increased community temporal stability under clipping. Species variance increases with species abundance; therefore, community stability is increased.

In the NC‐NF, MC‐NF, and HC‐NF communities, asynchrony effect and portfolio effect have major effects on community temporal stability. When community species diversity significantly decreases under disturbance (such as fertilization), the stability of dominant species will increase and stabilize ecosystem functioning (Yang et al., 2011) and community dominance will substantially decline after fertilization (Zhou et al., 2011). Therefore, the asynchrony effect and portfolio effect are deemed major mechanisms fostering community temporal stability under varying fertilization conditions.

Clipping increased community diversity and temporal stability, which explains the positive correlation we uncovered between them. In addition, this positive correlation was maintained by the portfolio effect in communities, yet fertilization reduced community diversity but strengthened community temporal stability. This result is mainly because while it decreases diversity, fertilization also simultaneously increases community‐level dominance and increased stability of dominant species promotes community temporal stability. Disturbance factors are external drivers of biodiversity and community dynamics, of which clipping, when viewed as a disturbance, is a candidate driver of biodiversity and stability relationships.

## 5 CONCLUSIONS

Clipping and its interaction with fertilization treatments significantly increased the temporal stability of plant communities in alpine meadow on the Tibetan. Clipping mainly increases community temporal stability through the asynchrony effect. In clipping and fertilization communities, the primary maintenance mechanisms of ecological important are the asynchrony effect and portfolio effect. Furthermore, the asynchrony effect, portfolio effect, and facilitation interactions occurred in all six different communities in this study. Finally, both positive and negative correlations can arise between diversity and community stability, which is mainly maintained by the asynchrony and portfolio effects.

## CONFLICT OF INTEREST

The corresponding author declares on behalf of authors that there is no conflict of interest to disclose.

## AUTHOR CONTRIBUTION

**Ting Wang:** Conceptualization (equal); Methodology (equal); Validation (equal); Writing‐original draft (lead). **Chenglong Guo:** Conceptualization (equal); Methodology (equal); Validation (equal); Writing‐original draft (equal). **Silin Sang:** Data curation (equal); Investigation (equal); Validation (equal). **Yiting Liu:** Data curation (equal); Investigation (equal); Validation (equal). **Gang Liu:** Methodology (equal); Software (equal); Supervision (equal). **Desheng Qi:** Software (equal). **Zhihong Zhu:** Conceptualization (equal); Methodology (equal); Supervision (lead).

## Supporting information

Supplementary Material

## Data Availability

Effects of clipping and fertilization treatments on the temporal stability of meadow plant communities in this paper were deposited in dryad dataset. https://doi.org/10.5061/dryad.9zw3r22fv.
