# Supplementary material for: Temporal stability and maintenance mechanisms of alpine meadow communities under clipping and fertilization
Source: Ecol Evol. 2021 Oct 28;11(22):15545–55. doi: 10.1002/ece3.8128 (PMC8601914; doi:10.1002/ece3.8128)
Supplement: Supplementary file 1 — Supplementary Material [file ECE3-11-15545-s001.docx]

TABLE S1 Regression analysis for log(*σ*^2^) vs. log(*m*) to six different meadow communities

| **Communities** | **Regression equations, *y*: log(*σ*^2^); x: log(*m*)** | ***R*^2^** | ***F*-test** | ***P*** | **m, n** |
| --- | --- | --- | --- | --- | --- |
| NC-NF | *y* = 1.552*x* − 0.104 | 0.918 | 1083.548 | < 0.001 | 1, 97 |
| NC-F | *y* = 1.616*x* + 0.041 | 0.931 | 1211.017 | < 0.001 | 1, 90 |
| MC-NF | *y* = 1.683*x* + 0.033 | 0.921 | 1071.782 | < 0.001 | 1, 92 |
| MC-F | *y* = 1.733*x* + 0.171 | 0.948 | 1636.742 | < 0.001 | 1, 90 |
| HC-NF | *y* = 1.545*x* − 0.257 | 0.925 | 1132.028 | < 0.001 | 1, 92 |
| HC-F | *y* = 1.582*x* − 0.073 | 0.958 | 2075.94 | < 0.001 | 1, 91 |

*Note:* m, degrees of freedom for the treatment; n, degrees of freedom for the error. The abbreviations NC-NF, NC-F, MC-NF, MC-F, HC-NF, and HC-F indicate the different treatments of no clipping-no fertilization, no clipping-fertilization, moderate clipping-no fertilization, moderate clipping-fertilization, heavy clipping-no fertilization, and heavy clipping-fertilization, respectively. *R*^2^, adjusted coefficient of determination.

TABLE S2 Regression analysis for CV_Dom_ vs. SR to six different meadow communities

| **Communities** | **Regression equations, *y*: *CV*_Dom_; *x*: *SR*** | ***R*^2^** | ***F*-test** | ***P*** | **m, n** |
| --- | --- | --- | --- | --- | --- |
| NC-NF | y = 18.102*x* + 53.293 | 0.286 | 6.412 | 0.022 | 1, 16 |
| NC-F | y = −3.673*x* + 60.350 | 0.036 | 0.594 | 0.452 | 1, 16 |
| MC-NF | y = −6.919*x* + 79.297 | 0.211 | 4.276 | 0.055 | 1, 16 |
| MC-F | y = 7.607*x* + 59.383 | 0.268 | 5.852 | 0.028 | 1, 16 |
| HC-NF | y = 0.157*x* + 63.244 | < 0.001 | 0.005 | 0.946 | 1, 16 |
| HC-F | y = 2.825*x* + 55.371 | 0.016 | 0.255 | 0.621 | 1, 16 |

*Note:* m, degrees of freedom for the treatment; n, degrees of freedom for the error. Abbreviations NC-NF, NC-F, MC-NF, MC-F, HC-NF, and HC-F indicate the different treatments of no clipping-no fertilization, no clipping-fertilization, moderate clipping-no fertilization, moderate clipping-fertilization, heavy clipping-no fertilization, and heavy clipping-fertilization, respectively; *R*^2^, adjusted coefficient of determination.

TABLE S3 Regression analysis for CV_Dom_ vs. CV_Com_ to six different communities

| **Communities** | **Regression equations, *y*: *CV*_Com_; *x*: *CV*_Dom_** | ***R*^2^** | ***F*-test** | ***P*** | **m, n** |
| --- | --- | --- | --- | --- | --- |
| NC-NF | *y* = −0.043*x* + 0.301 | 0.029 | 0.479 | 0.499 | 1, 16 |
| NC-F | *y* = −0.079*x* + 0.369 | 0.067 | 1.14 | 0.301 | 1, 16 |
| MC-NF | *y* = −0.013*x* + 0.174 | 0.009 | 0.147 | 0.706 | 1, 16 |
| MC-F | *y* = −0.027*x* + 0.168 | 0.062 | 1.057 | 0.319 | 1, 16 |
| HC-NF | *y* = +0.006*x* + 0.209 | 0.001 | 0.013 | 0.912 | 1, 16 |
| HC-F | *y* = +0.058*x* + 0.132 | 0.024 | 0.385 | 0.544 | 1, 16 |

*Note:* m, degrees of freedom for the treatment; n, degrees of freedom for the error. Abbreviations for NC-NF, NC-F, MC-NF, MC-F, HC-NF, and HC-F indicates the different treatments of no clipping-no fertilization, no clipping-fertilization, moderate clipping-no fertilization, moderate clipping-fertilization, heavy clipping-no fertilization, and heavy clipping-fertilization, respectively; *R*^2^, adjusted coefficient of determination.


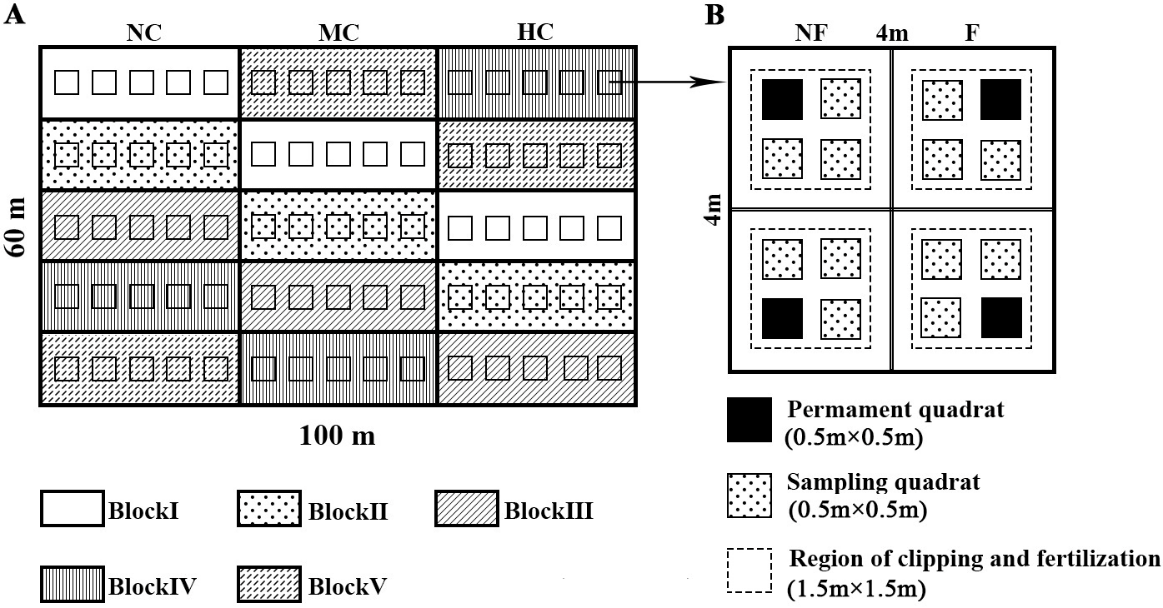
FIGURE S1 Design of plots (a) and layout of subplots (b). Roman capitals I, II, III, IV, and V represent five experiment blocks; NC, MC, and HC indicate noclipping, moderate clipping, and heavy clipping treatments, respectively; F and NF indicate fertilization and no fertilization treatments.
